# Editorial: New Drug Targets for Proteotoxicity in Cardiometabolic Diseases

**DOI:** 10.3389/fphys.2021.745296

**Published:** 2021-09-16

**Authors:** Jun Ren, Xin Wang, Yingmei Zhang

**Affiliations:** ^1^Department of Cardiology and Shanghai Institute of Cardiovascular Diseases, Zhongshan Hospital, Fudan University, Shanghai, China; ^2^Department of Laboratory Medicine and Pathology, University of Washington, Seattle, WA, United States; ^3^Faculty of Biology, Medicine and Health, The University of Manchester, Manchester, United Kingdom

**Keywords:** non-coding RNA, ischemia, metabolic, drug target, cardiometabolic

We are honored to serve as lead editors for this Frontier theme issue of “*New drug targets for proteotoxicity in cardiometabolic diseases*.” Our enthusiasm for this special topic rooted from the recent surge of novel medications and therapeutic modalities in cardiometabolic diseases in particular obesity, diabetes mellitus, insulin resistance, hypertension, ischemic/reperfusion injury and myocardial infarction (Ceylan-Isik et al., 2008; Ren and Zhang, 2018; Zhang et al., 2018; Aguilar-Ballester et al., 2021). Although development of medical technology has significantly advanced clinical diagnosis and management for cardiometabolic diseases, the ever-rising risks of metabolic diseases impose a main threat for health care in the 21st century (Onat, 2011; Kaur, 2014; Ren and Anversa, 2015; Zhang et al., 2018). Up-to-date, a number of theories have been suggested for the etiology of cardiometabolic anomalies including disturbed glucose/lipid metabolism, endoplasmic reticulum (ER) stress, oxidative and nitrosative/nitrative stress, mitochondrial injury, inflammation, and programmed cell death (apoptosis, necroptosis, pyroptosis, and ferroptosis) (Ren and Kelley, 2009; Ren et al., 2010, 2021; Onat, 2011; Ceylan-Isik et al., 2013; Kaur, 2014; Ren and Anversa, 2015). More evidence has noted an essential role for proteotoxicity or protein quality control in the pathogenesis of cardiometabolic diseases (Sandri and Robbins, 2014; Zhang et al., 2018). In general, protein quality control is governed by ubiquitin-proteasome system (UPS) and autophagy-lysosomal systems, two major albeit distinct machineries for degradation and removal of damaged and/or long-lived intracellular macromolecules (nucleic acids, proteins, carbohydrates, lipids) and organelles (e.g., mitochondria, ribosomes, peroxisomes, and ER) (Zhang et al., 2018; Ren et al., 2021). Interruption of UPS and autophagy evokes detrimental proteotoxic sequalae and is drawing much attention in the pathophysiology and pharmacotherapies in cardiometabolic diseases (Sandri and Robbins, 2014; Wang and Wang, 2014). In this context, this issue tackles some of the burning issues in cardiometabolic diseases with a particular focus on proteotoxicity and protein quality control.

In this issue, Li C. et al. discussed the role of Farnesoid X receptor (FXR) agonists as a possible target for the management of cardiometabolic diseases (Li C. et al.). FXR, a metabolic nuclear receptor, is mainly turned on by primary bile acids (BAs) such as chenodeoxycholic acid, cholic acid and synthetic agonists such as obeticholic acid. Recent evidence suggests that FXR agonists help to maintain regulation of cholesterol, lipid metabolism, glucose metabolism, and intestinal microorganism, with promises in the management of cardiometabolic diseases (Li C. et al.). Next, Tao et al. analyzed co-expression module genes from GSE18897 and GSE47022 to reveal 6 upregulated genes. These authors then validated upregulated levels of elongation of very-long-chain fatty acids protein 4, matrix metalloproteinase-8, and interleukin-33 in peripheral bloods from patients with obesity heart diseases. Given the poorly defined molecular machineries underneath obesity cardiomyopathy, findings from this integrated bioinformatics analysis favored a role for these hub genes as potential biomarkers for diagnosis and therapeutic targets in obesity heart diseases (Tao et al.). In this theme issue, Yang et al. discussed the benefits of antidiabetics metformin in the management of diabetic cardiomyopathy. These authors found that metformin activates prokineticin 2 (PK2), a small-molecule secreted protein governing survival and angiogenesis, to mediate Akt/GSK3β signaling, improve cardiac function and alleviate apoptosis in diabetic mice (Yang et al.). In this theme issue, Tian et al. provided insights into the complex interplay between histone acetyltransferases (HATs) and histone deacetylases (HDACs), two enzyme families controlling biological processes of histone acetylation and deacetylation, in the regulation of skeletal muscle physiology and energy homeostasis during exercise. Balance between these two enzymes plays a major role in mitochondrial remodeling, insulin sensitivity, on/off metabolic fuel switching and physiological homeostasis of skeletal muscles (Tian et al.). Type 2 diabetic patients usually display high levels of metabolic products such as trimethylamine N-oxide (TMAO) associated with gut dysbiosis. Here Steinke et al. discussed the value of TMAO levels in reference to cardiovascular risk. Next, Cao et al. examined levels of circulating ceramide in 761 patients with comorbid acute coronary syndrome (ACS) and type 2 diabetes mellitus, and noted that circulating ceramides are correlated with the risk of ACS-type 2 diabetes comorbidity. These innovative results suggested the utility of ceramide levels in the risk assessment of ACS- diabetes mellitus comorbidity (Cao et al.). Next, Schubert et al. summarized the possible cardiac benefits of new antidiabetic agents, including sodium-glucose cotransporter 2 (SGLLT2) inhibitor and glucagon-like peptide-1 receptor agonist, dipeptidyl peptidase-4 (DPP) inhibitor and metformin in patients with high cardiovascular risk with or without type 2 diabetes (Schubert et al.).

Myocardial ischemia injury and acute myocardial infarction remain the leading causes of mortality in patients with cardiovascular diseases (Zhang and Ren, 2014, 2016; Ren and Zhang, 2017). In this theme issue, Man et al. tackled the role of Shank3 in post-infarction cardiac dysfunction. They found that Shank3 knockout aggravated, while Shank3 overexpression alleviated, cardiac dysfunction following myocardial infarction. These findings revealed a novel role for Shank3 as a new therapeutic target for cardiac dysfunction following myocardial infarction through induction of autophagy (Man et al.). Next, Fu et al. succinctly dissected the compelling roles for melatonin in acute myocardial infarction. These authors elaborated the current well-perceived mechanisms underneath beneficial effects of melatonin against oxidative stress, promoting autophagic repair of cells, regulating immune and inflammatory responses, engaging mitochondrial function, and relieving ER stress, in the pathology of myocardial infraction injury (Fu Z. et al.). Microtubule and mitochondrial dysfunction are involved in cardiovascular disease etiology, including cardiac remodeling, heart failure, and hypoxic/ischemic injury. In this issue, Li L. et al. discussed MAP4 phosphorylation in microtubule instability. Phosphorylation of MAP4 triggers mitochondrial apoptosis, en route to cardiac injury, denoting a novel role for MAP4 as a candidate in cardiovascular pathologies (Li L. et al.). Next, Liu M. et al. discussed the therapeutic options for hypoxia-related cardiovascular disease. Hypoxia-inducible factor (HIF) is turned on in hypoxia to modulate diverse target genes. The authors recapitulated pathophysiology of hypoxic injury in cardiovascular disease, and provided convincing evidence for HIF-evoked cardioprotective signaling (Liu M. et al.). Cold stress is often associated with ischemia heart injury involving disturbed endothelin (ET-A) receptor, ER stress, transient receptor potential (TRP) vanilloid, mitochondrial damage. Here Kong et al. discussed various aspects of cold stress-induced cardiac injury, in particular dysregulated autophagy (Kong et al.). Next, Gao et al. introduced structural and functional characteristics of TRPA1 in cardiovascular diseases. They discussed evidence where TRPA1 is correlated to cardiovascular disease risk factors (Gao et al.). Exercising evokes benefits on heart failure with preserved ejection fraction (HFpEF). Then, Liu J. et al. designed a device to perform early passive leg movement (ePLM) and noted benefit of ePLM in alleviating high salt diet-induced HFpEF through inhibition of fibrosis in a TGF-β1/Smad3-dependdent manner in conjunction to Akt/eNOS signaling activation. These findings implied the benefit of ePLM as a novel non-pharmacological approach for HFpEF (Liu J. et al.). Chronic inflammation is part of ischemic heart disease process with overt atherosclerosis. Due to overwhelmed accumulation of monocytes/macrophages within arterial plaques, monocytes/macrophages are deemed critical cellular targets in the management of atherosclerosis. Many long non-coding RNAs (LncRNAs) were found to exert regulatory roles on metabolism and plasticity in macrophages, consequently stimulating or inhibiting atherosclerotic inflammation. Next, Ma et al. provided a balanced viewpoint of lncRNAs in macrophage biology, pathogenesis and therapeutics of atherosclerosis (Ma et al.). Furthermore, Chakafana et al. have carefully assessed the role of heat shock proteins in peripartum cardiomyopathy, and explored the possibility of given heat shock proteins as novel candidate biomarkers and drug targets in peripartum cardiomyopathy (Chakafana et al.). Next, Amin and team discovered a cardioprotective property of an orally active selenium based compound phenylaminoethyl selenides against doxorubicin cardiomyopathy possibly through preserving the stability of the iron-sulfur cluster biogenesis protein Frataxin (FXN) (Fu X. et al.). Moreover, Zhang et al. suggest that STAT6 plays a vital role in isoproterenol-induced β1-AR overactivation and inflammatory response, en route to cardiac fibrogenesis. Their findings denoted a novel role for STAT6 as a promising target against myocardial fibrosis and heart failure (Zhang et al.). Next, Njegic et al. addressed the molecular changes to proteins associated with Ca^2+^-handling and -signaling. These authors made an in-depth analysis on these potential novel therapeutic targets from the perspective of both pre-clinical and, clinical settings (Njegic et al.). In this series of collection, Ding et al. reported a novel mechanism where angiotensin (AngII) evokes endothelial dysfunction likely via AT1R-mediated protein phosphatase 2A (PP2A) activation to suppress endothelial nitric oxide synthase (eNOS) phosphorylation *via* Nox/ROS pathway (Ding et al.).

Given the advances in science and technology, non-coding (LncRNA) or microRNAs (miRNA) are considered as important regulators of cellular processes, and serve as an important platform for therapeutic regimen of cardiovascular disease. Nonetheless, many questions remain unanswered for LncRNA and miRNA, such as consequences of targeting, refinement of targeting systems. Here Collins et al. summarized the level of genomic regulation strategy for the treatment of cardiovascular disease (Collins et al.). Furthermore, Yan et al. identified COX-2 and related microRNA as possible biomarkers for non-ischemic heart failure. These authors analyzed genomic and transcription information from peripheral blood mononuclear cells of heart failure patients and found enrichment of prostaglandin-endoperoxide synthase 2 (PTGS2, or cyclooxygenase-2 - COX-2), as well as its related micro RNAs including miR-1297 and miR-4649-3p as potential biomarkers for non-ischemic heart failure. These authors noted overtly elevated plasma COX-2 and miR-4649-3p I conjunction with downregulated plasma miR-1297. Moreover, these authors suggested that miR-4649-3p possessed high predictive power for non-ischemic heart failure (Yan et al.). Although this theme issue did not touch too much on clinical trials, Yang et al. did a cross-sectional survey of 241 trials conducted on stem cell therapy. These authors shared their “disappointment” on the outcome of these registered clinical applications (Yang et al.).

Although this theme issue has shed some insights toward a more thorough knowledge on possible drug targets in cardiometabolic diseases, we regret to say we have failed in a large part identifying specific target(s) or drug(s) on autophagy or mitophagy molecules (the benchmarks for proteotoxic issue). This may reflect the overall challenge and difficulty in drug development targeting autophagy/mitophagy. Nonetheless, the recent advances in computer-based biological modeling and bioinformatic system should help us to better achieve desirable chemicals to target a given protein or signaling molecule in cardiometabolic diseases. We hope that this theme issue will aid to unveil novel therapeutic targets to launch better intervention regimens for cardiometabolic diseases.

## Author Contributions

JR, XW, and YZ contributed to drafting and editing of this editorial. All authors contributed to the article and approved the submitted version.

## Conflict of Interest

The authors declare that the research was conducted in the absence of any commercial or financial relationships that could be construed as a potential conflict of interest.

## Publisher's Note

All claims expressed in this article are solely those of the authors and do not necessarily represent those of their affiliated organizations, or those of the publisher, the editors and the reviewers. Any product that may be evaluated in this article, or claim that may be made by its manufacturer, is not guaranteed or endorsed by the publisher.

## References

[B1] Aguilar-Ballester M. Hurtado-Genoves G. Taberner-Cortes A. Herrero-Cervera A. Martinez-Hervas S. Gonzalez-Navarro H. (2021). Therapies for the treatment of cardiovascular disease associated with type 2 diabetes and dyslipidemia. Int. J. Mol. Sci. 22:20660. 10.3390/ijms22020660 33440821 PMC7826980

[B2] Ceylan-Isik A. F. Fliethman R. M. Wold L. E. Ren J. (2008). Herbal and traditional Chinese medicine for the treatment of cardiovascular complications in diabetes mellitus. Curr. Diabetes Rev. 4, 320–328. 10.2174/157339908786241142 18991600

[B3] Ceylan-Isik A. F. Kandadi M. R. Xu X. Hua Y. Chicco A. J. Ren J.. (2013). Apelin administration ameliorates high fat diet-induced cardiac hypertrophy and contractile dysfunction. J. Mol. Cell Cardiol. 63, 4–13. 10.1016/j.yjmcc.2013.07.002 23859766

[B4] Kaur J. (2014). A comprehensive review on metabolic syndrome. Cardiol. Res. Pract. 2014:943162. 10.1155/2014/943162 24711954 PMC3966331

[B5] Onat A. (2011). Metabolic syndrome: nature, therapeutic solutions and options. Exp. Opin. Pharmacother. 12, 1887–1900. 10.1517/14656566.2011.585462 21756201

[B6] Ren J. Anversa P. (2015). The insulin-like growth factor I system: physiological and pathophysiological implication in cardiovascular diseases associated with metabolic syndrome. Biochem. Pharmacol. 93, 409–417. 10.1016/j.bcp.2014.12.006 25541285

[B7] Ren J. Bi Y. Sowers J. R. Hetz C. Zhang Y. (2021). Endoplasmic reticulum stress and unfolded protein response in cardiovascular diseases. Nat. Rev. Cardiol. 18, 499–521. 10.1038/s41569-021-00511-w 33619348

[B8] Ren J. Kelley R. O. (2009). Cardiac health in women with metabolic syndrome: clinical aspects and pathophysiology. Obesity. 17, 1114–1123. 10.1038/oby.2009.8 19214173

[B9] Ren J. Pulakat L. Whaley-Connell A. Sowers J. R. (2010). Mitochondrial biogenesis in the metabolic syndrome and cardiovascular disease. J. Mol. Med. 88, 993–1001. 10.1007/s00109-010-0663-9 20725711 PMC4319704

[B10] Ren J. Zhang Y. (2017). Editorial: new therapetic approaches in the management of ischemia reperfusion injury and cardiometabolic diseases: opportunities and challenges. Curr. Drug Targets. 18, 1687–1688. 10.2174/138945011815171019092703 29070009

[B11] Ren J. Zhang Y. (2018). Targeting autophagy in aging and aging-related cardiovascular diseases. Trends Pharmacol. Sci. 39, 1064–1076. 10.1016/j.tips.2018.10.005 30458935 PMC6251315

[B12] Sandri M. Robbins J. (2014). Proteotoxicity: an underappreciated pathology in cardiac disease. J. Mol. Cell. Cardiol. 71, 3–10. 10.1016/j.yjmcc.2013.12.015 24380730 PMC4011959

[B13] Wang C. Wang X. (2014). The interplay between autophagy and the ubiquitin-proteasome system in cardiac proteotoxicity. Biochim. Biophys. Acta 1852, 188–194. 10.1016/j.bbadis.2014.07.028 25092168 PMC4277934

[B14] Zhang Y. Ren J. (2014). Targeting autophagy for the therapeutic application of histone deacetylase inhibitors in ischemia/reperfusion heart injury. Circulation 129, 1088–1091. 10.1161/CIRCULATIONAHA.113.008115 24396040

[B15] Zhang Y. Ren J. (2016). Bridging the gap, facing the challenge-the 26(th) great wall international congress of cardiology (GW-ICC). Cardiovasc. Diagn. Ther. 6, 97–100. 10.3978/j.issn.2223-3652.2016.01.01 26885499 PMC4731590

[B16] Zhang Y. Whaley-Connell A. T. Sowers J. R. Ren J. (2018). Autophagy as an emerging target in cardiorenal metabolic disease: from pathophysiology to management. Pharmacol. Ther. 191, 1–22. 10.1016/j.pharmthera.2018.06.004 29909238 PMC6195437

